# Cell Integration with Electrospun PMMA Nanofibers, Microfibers, Ribbons, and Films: A Microscopy Study

**DOI:** 10.3390/bioengineering6020041

**Published:** 2019-05-09

**Authors:** Daniel P. Ura, Joanna E. Karbowniczek, Piotr K. Szewczyk, Sara Metwally, Mateusz Kopyściański, Urszula Stachewicz

**Affiliations:** 1 International Centre of Electron Microscopy for Materials Science, Faculty of Metals Engineering and Industrial Computer Science, AGH University of Science and Technology, 30-059 Krakow, Poland; urad@agh.edu.pl (D.P.U.); jkarbow@agh.edu.pl (J.E.K.); pszew@agh.edu.pl (P.K.S.); metwally@agh.edu.pl (S.M.); 2 Faculty of Metals Engineering and Industrial Computer Science, AGH University of Science and Technology, 30-059 Krakow, Poland; mkopys@agh.edu.pl

**Keywords:** filopodia, osteoblast, fibers, ribbons, electrospinning, film, PMMA

## Abstract

Tissue engineering requires properly selected geometry and surface properties of the scaffold, to promote in vitro tissue growth. In this study, we obtained three types of electrospun poly(methyl methacrylate) (PMMA) scaffolds—nanofibers, microfibers, and ribbons, as well as spin-coated films. Their morphology was imaged by scanning electron microscopy (SEM) and characterized by average surface roughness and water contact angle. PMMA films had a smooth surface with roughness, *R_a_* below 0.3 µm and hydrophilic properties, whereas for the fibers and the ribbons, we observed increased hydrophobicity, with higher surface roughness and fiber diameter. For microfibers, we obtained the highest roughness of 7 µm, therefore, the contact angle was 140°. All PMMA samples were used for the in vitro cell culture study, to verify the cells integration with various designs of scaffolds. The detailed microscopy study revealed that higher surface roughness enhanced cells’ attachment and their filopodia length. The 3D structure of PMMA microfibers with an average fiber diameter above 3.5 µm, exhibited the most favorable geometry for cells’ ingrowth, whereas, for other structures we observed cells growth only on the surface. The study showed that electrospinning of various scaffolds geometry is able to control cells development that can be adjusted according to the tissue needs in the regeneration processes.

## 1. Introduction

Tissues are the functional basic units in the body, built of cells surrounded by the extracellular matrix (ECM). To grow tissues in the laboratory conditions, a supportive structure in the form of a scaffold must be provided. Therefore, interactions of cells with the designed scaffolds are the key to a proper tissue development and regeneration processes [1]. The matrix, or scaffold, does not only support the cells but also helps in the cells’ signaling pathways. Understanding how individual cells respond and interact with their surroundings allows the development of scaffold geometry to enhance the processes of tissue regeneration [2]. Therefore, engineering of surfaces to mimic ECM is critical in biomaterial science and all tissue regeneration applications, to enable assembling of larger and more complex tissues. To build effective tissues, cells need to first develop a suitable morphology and physical features, such as filopodia, in the in vitro conditions [3]. Cells reorganize by interactions with material properties, such as topography [4], mechanical strength [5], and surface potential [6,7,8]. Moreover, they adjust the shape and morphology to a surface geometry, thus, the design of the scaffold is crucial in controlling cells behavior, development, and proliferation [9,10].

Electrospinning is one of the most commonly used methods to produce nanofibers, microfibers, ribbons, and other shapes for mimicking ECM [11], which are often evaluated as scaffolds for tissue engineering [12,13]. The fibrous scaffolds also have the advantage of a large surface-area-to-volume ratio [14]. Additionally, surface properties, various modifications, and functionalizations have been widely studied [15]. Plasma-treated poly(L-lactic acid) electrospun fibers were shown to enhance the initial cells adhesion, due to the increased surface content of oxygen-containing polar groups [16]. In another study [17], fibers functionalized by the incorporation of hydroxyapatite nanoparticles had an increased tensile strength, compared to the unmodified scaffolds, which resulted in an improved expression of actin filaments and alkaline phosphatase activity of osteoblasts. The organization and alignment of fibers or other features, thus generally the geometry, is able to guide the direction of cell growth [18,19,20] and migration [21,22]. Electrospun nanofibrous scaffolds are most often considered as 2D structures hampering cell infiltration, due to the small pores sizes [23]. Pores in electrospun mats, which are responsible for 3D integration of cells within scaffolds, are strongly related to fibers diameter, processing parameters, and the collector type [24]. Several strategies have been proposed to increase the pores dimensions within the electrospun mats, to improve cells infiltration. One of them is formation of hybrid structures containing nano-/micro-fibers, which was shown to improve the cells ingrowth [25]. In such constructs, presence of microfibers allowed the cells to infiltrate the scaffold, due to the presence of large pores, while the nanofibers facilitated cell spreading. Another approach to optimize the porosity and pores diameters in electrospun scaffolds is by proper selection of collector design. Vaquette et al. [26], applied the following collectors—flat with different diameter of holes, mesh/wire, star, and ladder collectors, and showed that the diameter of the pores was increased from 5 µm to above 15 µm, resulting in an improved cells infiltration for scaffolds with larger pores. Fiber to fiber distances could also be increased by low-temperature electrospinning [27]. Where ice crystals are formed in between fibers, during processing in cryogenic conditions, they are subsequently removed by freeze-drying, leaving large voids in the scaffold structure, and facilitating cells infiltration.

Among many polymers applied in tissue engineering, we decided to focus this fundamental study on poly(methyl methacrylate) (PMMA), as it is a highly biocompatible material commonly used in medical applications for contact lenses or as bone cement [28], and has been extensively studied in the form of electrospun scaffolds for regenerative medicine [17]. So far, the effect of the PMMA fiber diameter and orientation, as well as smooth films on morphology, alignment, and migration of human dermal fibroblasts, have been studied [29]. For Schwann cells, the aligned PMMA fibers supported cell expansion and growth, along the main axes, while maintaining their viability [30], thus presenting promising results towards neuritis regeneration. Xing et al. [17] studied the PMMA scaffolds with incorporated hydroxyapatite (HA) for bone tissue regeneration and mineralization. A few studies have used PMMA scaffolds with surface modifications [31], with collagen coating [32], proteins [33] or blended with polycaprolactone (PCL) [34], to improve the wettability of the scaffolds, and therefore, the cell proliferation and biocompatibility [35]. The improved wettability of scaffolds can be achieved via protein-mediated cell adhesion, thus, the hydrophobic properties of electrospun fibers are able to promote cells integration and proliferation [7,8]. For PMMA, a film contact angle of 76.6° was reported, whereas electrospun PMMA mats had significantly increased hydrophobicity, depending on the processing parameters, the contact angle was in the range of 100–147° [36]. 

In our studies, we analyzed cell integration, including the development and extension of filopodia with electrospun PMMA scaffolds, in the form of nanofibers, microfibers, and ribbons, and compared them with the spin-coated films. All PMMA samples represented different surface architecture and scaffold designs, without any chemical modifications. We performed a detailed scanning electron microscopy (SEM) study of filopodia attachment to the substrates made of the same material, as we only changed the geometry of the scaffolds and the surface roughness. Cells anchoring were also verified to the above mentioned substrates. This study allowed to observe how cells interact and integrate, and also change their shape and morphology, according to the various designs of scaffolds and surfaces.

## 2. Materials and Methods

### 2.1. Materials

PMMA with two different molecular weights, *M*_w1_ = 150,000 g·mol^−1^ and *M*_w2_ = 350,000 g mol^−1^ and solvents—N, N-dimethylformamide (DMF) and formic acid—were purchased from Sigma Aldrich, Dorsen, UK. The polymer solutions were stirred for 2.5 h at 55 °C, on a heating plate (IKA RCT basic, Staufen, Germany), to obtain a transparent solution indicating a completely dissolved polymer powder. PMMA films were spin-coated by depositing of 3 mL droplets of polymer solution on glass coverslips (18 × 18 mm) and applying a rotation speed of 2,500 rpm for 20 s. After spin-coating, the film was dried at room temperature for 48 hours and stored in a Petri dish. The thickness of the film was approximately 0.3 mm. The details of polymer solutions for electrospinning and spin-coating preparation are listed in Table 1. The experimental setting and polymer *M*_w_ were selected according to previous studies [37,38], to obtain the desired PMMA scaffolds.

### 2.2. Electrospinning and Spin-Coating

Electrospinning of PMMA fibers and ribbons was carried out using the apparatus EC–DIG with a climate upgrade system (IME Technologies, Waalre, the Netherlands), at a constant T = 25 °C and H = 40–45% in the environment chamber with other key parameters listed in Table 1. The deposition time of all electrospun samples was 30 min. The PMMA films were spin-coated (Ossila, L2001A3, Sheffield, UK) on glass slides, by depositing 3 mL droplets of the polymer solution, at a speed of 2,500 rpm for the 20 s. Films were dried at room temperature for 48 hours. All samples after preparation were stored in Petri dishes in a desiccator.

### 2.3. Surface Characterization: Roughness and Wetting

The optical profilometer (Vecco, WykoNT9300, Plantview, NY, USA) was used to obtain an average roughness parameter (*R*_a_) of the investigated surfaces, using the VSI (Vertical Scanning Interferometry mode—objective (20×), field-of-view multiplier (0.55×), and set-up parameters; size (640 × 480) and sampling (910.43 nm) as previously described [38]. *R*_a_ is defined as a roughness average and arithmetic mean of the absolute values of the surface departures from the mean plane.

Advancing water contact angles (*θ*) on electrospun PMMA nanofibers, microfibers, ribbons, and spin-coated films were measured using deionized water (Spring 5UV purification system, Hydrolab, Poland, γ = 72.2 mJm^−1^). A total of 3 μL volume droplets were pipetted onto the samples and, after 3 s from the water deposition, the image was taken using a DSLR camera (EOS 700D, Canon, Tokyo, Japan) with a macro lens (EF-S 60mm f/2.8 Macro USM). Experiments were carried out in the laboratory at T = 25 °C and H = 45%. The contact angles were measured using a drop shape analysis plug-in, in ImageJ (Fiji, version J1.46r., Würzburg, Germany). The average contact angle was calculated from measurements taken on 10 droplets, with the error based on the standard deviation.

### 2.4. Cell Culture 

Human osteoblast-like cell line MG63 (ECACC, Sigma-Aldrich, Dorset, UK) were cultured on 4 different morphologies of PMMA—nanofibers, microfibers, ribbons, and films, for 1 and 3 days, in constant T = 37 °C, H = 90% and 5% CO_2_ atmosphere. Prior cell culture for all scaffolds were sterilized by UV light, for 30 min, in a laminar flow cabinet. Cell culture media was based on Dulbecco’s Modified Eagle’s medium (DMEM), supplemented with 10% fetal bovine serum (FBS), non-essential amino acids, l-glutamine, penicillin/streptomycin (all reagent from Sigma Aldrich, Dorset, UK). A total of 2 mL of cells suspension in culture media of concentration 4 × 10^4^ cells·mL^−1^ was added per sample. Samples after incubation were washed three times with phosphate buffered saline (PBS) and fixed with 2.5% glutaraldehyde (Sigma Aldrich, Dorset, UK), for 1 h, in 4 °C. Subsequently, the samples were washed three times in PBS and air dried under the fume hood for the microscopy investigation.

### 2.5. Scanning Electron Microscopy (SEM)

**Figure 1:**
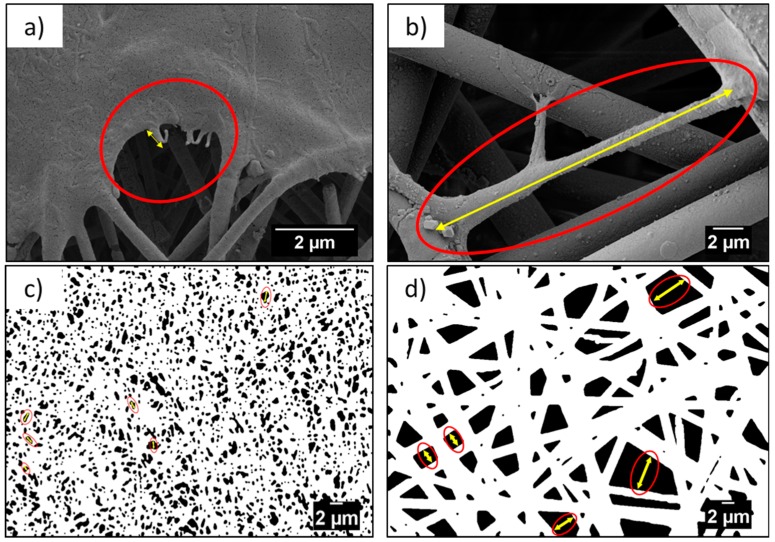
Example of measurement (**a**,**b**) cells’ filopodia, *L*_c_, and (**c**,**d**) distance measurement between fibers, *S*_f_, from the SEM micrographs between nanofibers and microfibers, respectively.

Morphology of the PMMA scaffolds and the films, as well as the cell attachments and morphology in contact with various geometries, was studied using SEM (ZEISS, Merlin Gemini II, Cologne, Germany), with a 5 nm gold layer coating applied by the rotary pumped coater (Quorum Technologies Ltd., Q150RS, Lewes, UK), prior to the investigation. Scaffolds were imaged with an accelerating voltage of 3 kV, 120 pA current, and a working distance ranging from 5 to 9 mm. From the SEM images, we measured the average diameter (*D*_f_) of fibers and ribbons, based on 100 measurements, using ImageJ, a diameter characterization tool—DiameterJ plugin [39]. The average distance between fibers (*S*_f_) was estimated based on 100 measurements for nano- and microfibers, using a length tool plugin for ImageJ. Additionally, the length of the cells’ filopodia (*L_c_*) was measured for 20 different filopodia. The schematic measurement method of *S*_f_ and *L*_c_ is presented in Figure 1. The error in all measurements from SEM images was based on the standard deviation.

## 3. Results and Discussion

### 3.1. Fibers and Films Morphology, Roughness, and Wetting Properties

**Figure 2:**
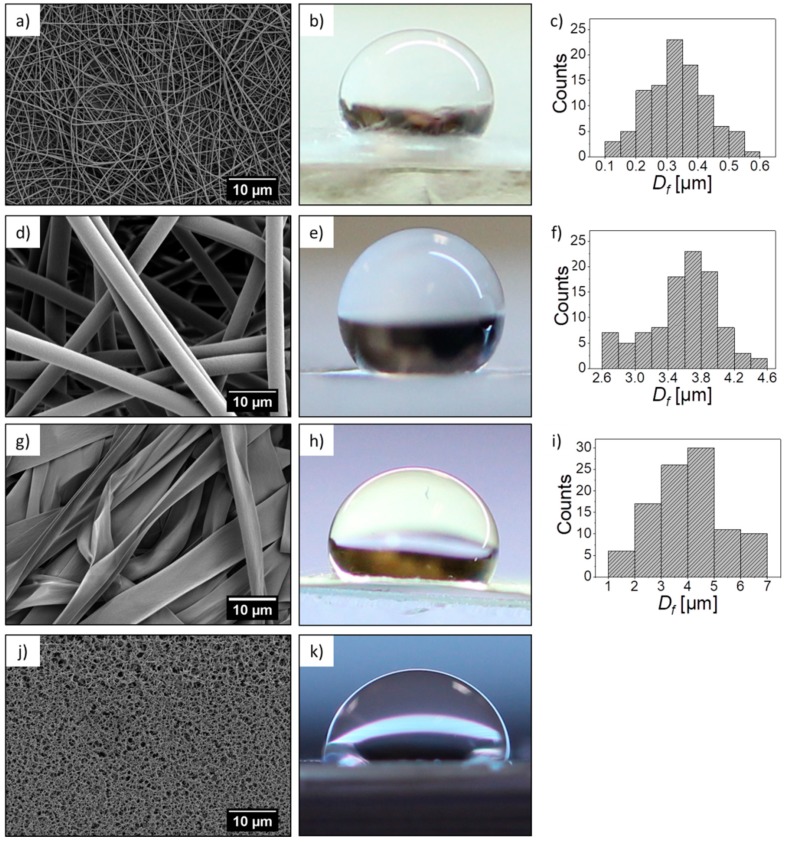
Poly(methyl methacrylate) (PMMA) (**a**) nanofibers, (**d**) microfibers, (**g**) ribbons, (**j**) and films SEM micrographs, and their corresponding representative images of the water droplet used for contact angle measurement (**b**,**e**,**h**,**k**), respectively, and (**c**,**f**,**i**) histograms showing the diameter of the fiber size distribution.

**Figure 3:**
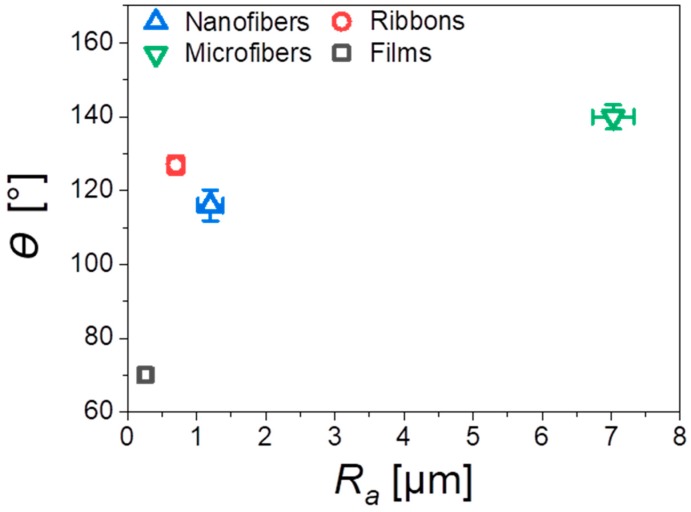
Water contact angles *θ* as a function of the average roughness, *R*_a,_ for all investigated PMMA scaffolds and film.

In this work, we investigated four different types of PMMA substrates—nanofibers, microfibers, and ribbons produced by electrospinning and spin-coated films; their morphologies and characteristic parameters are presented in Figure 2. The main difference between the scaffolds and films was the roughness, verified with the profilometry study, see Table 2. The average roughness, *R*_a_, measured for the flat films was 0.26 µm, and for the electrospun fibers, we observed an increased *R*_a_ with the highest value of 7.03 µm for the microfibers. In the case of the PMMA film, we noticed microporosity (Figure 2j), which was due to the very fast evaporation of solvent during the spin-coating [40]. Generally, the roughness of the electrospun mats was related to their average fiber diameter, typically, a larger diameter corresponded to a higher roughness [38]. Example of such observations were described for poly(lactic-co-glycolic acid) (PLGA) scaffolds [41]. Where the smooth films had an *R*_a_ of 0.42 µm, and for the electrospun fibrous mats, the bigger the fiber diameter, the higher roughness. PLGA scaffolds with a fiber diameter around 1 µm had an *R*_a_ of 1.46 µm and those with a fiber diameter around 6.5 µm, had an *R*_a_ of 5 µm. The average diameter of the nanofibers was 300 nm, for microfibers 3.6 µm, whereas the width of the ribbons reached almost 4 µm, as summarized in Table 2. Since the ribbons were mostly flat, they created a sort of layered structure where the neighboring fibers, to large extent, adhered to each other, which contributed to the relatively low roughness (*R*_a_ = 0.7 µm), for this type of scaffold. Moreover, the increase of roughness was responsible for the increase of the contact angle, up to 140° for microfibers, see Figure 3, showing a very high hydrophobicity of PMMA microfibers. The water contact angle on the PMMA film was 70°, indicating a hydrophilic behavior, which has been previously investigated [38]. In Figure 3, we clearly indicated the increase of contact angle with an increase of *R*_a_, as the air captured between the fibers is able to reduce the surface free energy of fibers compared to films. Therefore, the electrospun PMMA scaffolds were in the Cassie–Baxter regime of wetting [42]. The wetting properties of the PMMA samples, in relation to the diameter of fiber and roughness, have been previously investigated [38].

### 3.2. Cell Morphology and Integration with Scaffolds

**Figure 4:**
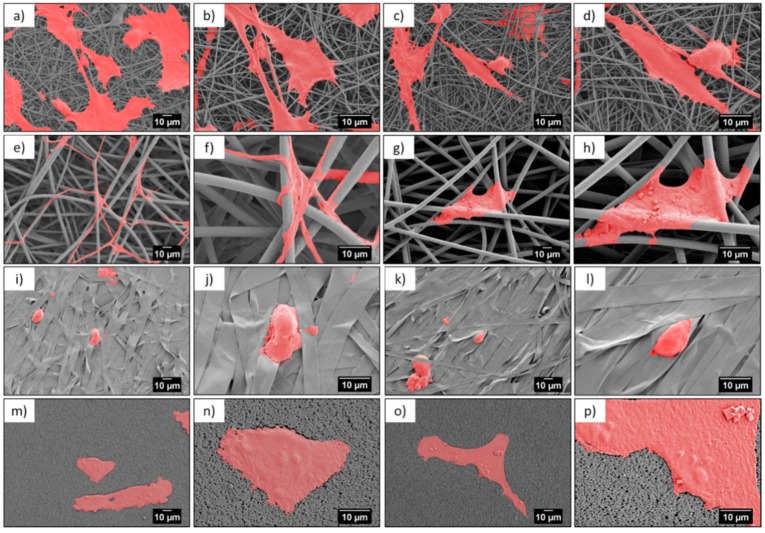
SEM micrographs of cells (colored in red) after 1 day in cell culture on PMMA scaffolds built of (**a**–**d**) nanofibers, (**e**–**h**) microfibers, (**i**–**l**) ribbons, and (**m**–**p**) film.

**Figure 5:**
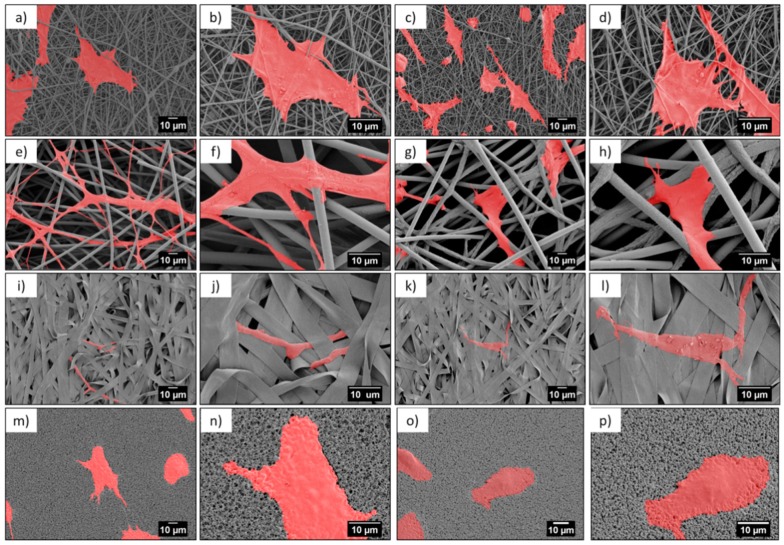
SEM micrographs of cells (colored in red) after 3 days in cell culture on PMMA scaffolds built of (**a**–**d**) nanofibers, (**e**–**h**) microfibers, (**i**–**l**) ribbons, and (**m**–**p**) film.

**Figure 6:**
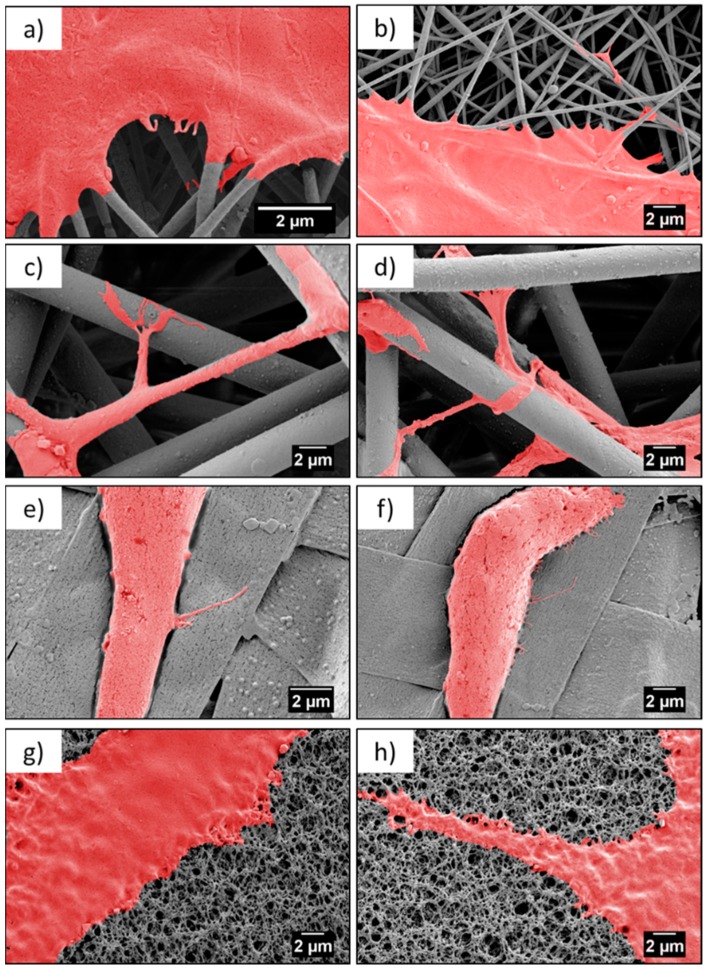
SEM micrographs showing cell attachment to a different type of scaffold, after three days—(**a**,**b**) nanofibers, (**c**,**d**) microfibers, (**e**,**f**) ribbons, and (**g**,**f**) film.

Microscopy investigation allowed us a direct observation of the cells’ morphology, according to the design of the PMMA-based scaffolds. Figure 4 and Figure 5 show the integration of the cell, after 1 and 3 days of the cell culture being presented, showed differences in cell shape and morphology. Within the first day, the cells were flattened and well-spread on nano- and microfibers, and films, whereas, on the ribbons they showed a spherical, slightly elongated shape. After one day, the cells observed on the ribbons were located on the individual pieces and aligned their morphology along it (Figure 4j,l). The width of the ribbons, around 4 µm, created a border for the cells spreading across the surface, thus, the cells rather elongated their shape along the individual ribbon than spreading beyond it. Additionally, a limited filopodia development was observed on the cells grown on ribbons, as demonstrated in Figure 4j,l; the average length of filopodia was below 0.5 µm. After three days of cell culture, the cells continued to integrate with the PMMA surfaces. In case of nanofibers, they flattened and spread, however, we observed an increased filopodia wrapping around individual fiber, for microfibers; higher magnification images can be found in Figure 6c,d and Table 2. Cells on the ribbons after three days, also started to flatten, as it was not observed after the first day of culture, thus, they needed more time to develop all features that allowed them to spread. The geometry of ribbon-based scaffolds was densely packed, as shown in Figure 2d, indicating more of a 2D structure than a 3D scaffold. In this case, small distances between ribbons limited the 3D integration, meaning that it caused a cells migration and ingrowth into the scaffold structure, as the wrapping of the cells filopodia on ribbons, was not observed. In terms of nano- and microfibers, the distance between the fibers, related to their diameter, affected cell behavior [12]. Cells on nanofibers were nicely spread on the top of the scaffolds, as the space between fibers (*S*_f_ of around 0.6 µm) also limited the cells ingrowth (Figure 6a,b), keeping their development only on the electrospun surface. Importantly, on the nanofibers, after three days in the cell culture, we observed very similar cell morphology and spreading, as on films (see Figure 5), even though the roughness and wettability differed significantly for these structures (see Table 2). The cell spreading, as well the filopodia length, was strongly related to the pore size. In the case of nanofibers, the pore size was in the submicron level, limited by the diameter of the fibers [43], therefore, the osteoblasts spreading was only on the surface of the electrospun mat. In the case of microfibers, due to the large fiber diameter, the distances between the fibers were up to a few microns, forcing the cells to bridge the gap between them, and to produce long filopodia, reaching nearly 20 µm, Figure 4 (the average *L*_c_ for microfibers was approximately 13 µm). The microfibers provided enough space for cell ingrowth into the 3D scaffold. Generally, based on the microscopy study, we could summarize that, for scaffolds with a small fiber diameter, and spaces between the fibers, as well as the 2D structures (namely, nanofibers, ribbons, and films), we noted cell spreading only on surfaces, whereas, microfibers created the beneficial environment for cell ingrowth into a 3D mesh. We expected a penetration of cells into the electrospun scaffold with microfibers, as it was related to the pore sizes and distances between the fibers. As previously reported for the PLGA scaffolds [15], after 4 days of osteoblasts incubation, the cell penetration was up to 7 µm, into the random fibers network, whereas, the aligned fiber cells were only spread on the top of scaffold.

Contact guidance of cells related to the properties of the substrate is an essential component in regulating cell migration, which is modulated by organized ECM proteins, among others [44]. The adhesion of osteoblasts to the PMMA scaffolds was critical for cells growth and focal adhesion contacts related to the protrusion of cell membranes, visualized by filopodia formation and cells morphology. Importantly, the elongation of the filopodia facilitated the osteoblast adhesion and migration [34,35]. The environment has an enormous ability to steer cell protrusion by organization of the actin filaments within the formed filopodia, which have the ability to sense the surface topography [45]. The spreading of cells is a dynamic process that involves the stretching and retracting of the filopodia in all directions, to explore the surrounding, thereby, controlling the filopodia length [46,47]. The geometry and sizes of the electrospun fibers transforms the morphology of the cells’ filopodia, according to the designed environment, to study cell behavior through an in vitro study [15,19]. The largest spread of cells was observed on nanofibers, but the longest filopodia had developed on the microfibers (*L*_c_ ≈ 13 µm). The less developed cells, meaning less flattened and spread, were on the ribbons and the shortest filopodia were observed on the films, in comparison to the microfibers, as presented in the SEM images on Figure 6. This study clearly indicated that microfiber-based scaffolds enhanced the filopodia formation and their arrangement. Notably, ribbons were not favorable surfaces for cells development, as the typical sizes of the osteoblasts were 20–30 µm but the cell sizes observed on the ribbons were much smaller, as shown in Figure 4, Figure 5 and Figure 6, in comparison to the cells attached to other fibrous structures or films.

## 4. Conclusions

The microscopy investigation of cell integration with PMMA scaffolds showed an increased surface roughness, related to the fiber diameter, which was the key to enhance the interactions of the cells with the microfibers. All electrospun samples exhibited a water contact angle above 115°. The highest hydrophobicity of the PMMA microfiber scaffolds, created due to the increased roughness, was not a barrier for cells adhesion. The microfiber scaffolds showed the best cells anchoring, in comparison to the nanofibers, ribbons, and films. Nanofiber-based samples were considered to be 3D scaffolds, however, the distances between the fibers limited the penetration of the cells into the fibrous scaffold. Therefore, the diameter of fiber exceeding 3.5 µm, as in the case of microfibers, was required to provide the right geometry and enough spacing for cell migration into the 3D scaffold structure, which was initiated by the filopodia attachments to the fibers underneath. In summary, nanofibers facilitated more cell spreading on the top of the surface, in comparison to the microfibers, where the cell filopodia could reach fibers deeper into the electrospun mat. 

We proved with high-resolution imaging that not only the geometry of the scaffold but also the size of the fibers and the distance between them was crucial in cell development for tissue engineering [19]. SEM imaging is a highly effective method to evaluate micron and sub-micron filopodia development, on fibrous scaffolds and films, allowing us to understand the cell–scaffold interactions for tissue engineering applications. Future study will focus on microfibers with a similar geometry of scaffold, using various polymers, to verify the optimal materials for enhancing osteoblasts development for tissue regeneration.

## Figures and Tables

**Table 1:** Details of solution preparation for electrospinning and spin-coating of poly(methyl methacrylate) (PMMA) including—molecular weight (*M*_w_), concentration w/w (*C*), solvents, and the rotation speed of stirrer (*V*_r_); the electrospinning parameters—voltage (*U*), distance needle to collector (*d*), flow rate (*Q*), and humidity (*H*), used to produce PMMA scaffolds with nanofibers, microfibers, and ribbons.

PMMA Scaffold	Solution Preparation				Electrospinning Parameters			
	*M*_w_ [g·mol^−1^]	Solvent	*C* [%]	*v*_r_ [rpm]	*U* [kV]	*d* [cm]	*Q* [mL·h^−1^]	*H* [%]
Nanofibers	150,000	Formic Acid: DMF 7:3	12	500	11	10	0.3	40
Microfibers	150,000	DMF	30	750	12	15	3	40
Ribbons	350,000	Formic Acid	12	500	13	15	0.6	45
Film	350,000	DMF	12	500	-	-	-	-

**Table 2:** The summary of all measurements performed on the PMMA samples and cells, including the average diameter of fiber (*D*_f_), average roughness (*R*_a_), the average distance between fibers (*S*_f_), and the water contact angle (*θ*), on all PMMA surfaces; and the filopodia length, after 3 days of cell culture (*L*_c_).

Scaffold/Film	*D_f_* [µm]	*S_f_* [µm]	*R_a_* [µm]	*θ* [⁰]	*L_c_* [µm]
Nanofibers	0.34 ± 0.10	0.59 ± 0.33	1.20 ± 0.18	116.0 ± 4.2	0.47 ± 0.21
Microfibers	3.59 ± 0.44	3.21 ± 1.98	7.03 ± 0.30	139.9 ± 3.3	12.96 ± 6.78
Ribbons	3.93 ± 1.37 *	-	0.70 ± 0.12	126.9 ± 2.4	-
Films	-	-	0.26 ± 0.07	70.0 ± 1.9	-

* In case of ribbons, the width of the fiber was measured.
